# Late-Onset Gastrointestinal Manifestations of Multiple Endocrine Neoplasia Type 2B (MEN2B): Diffuse Ganglioneuromatosis Causing Megacolon

**DOI:** 10.7759/cureus.98366

**Published:** 2025-12-03

**Authors:** Maheen Rana, Saddam Hussain, Samira Osman, Paul Anant

**Affiliations:** 1 General Surgery, Our Lady of Lourdes Hospital, Drogheda, Drogheda, IRL; 2 Pathology, Our Lady of Lourdes Hospital, Drogheda, Drogheda, IRL; 3 General Surgery, Portiuncula University Hospital, Ballinasloe, IRL

**Keywords:** chronic megacolon, ganglioneuromatosis, gi, megacolon, multiple endocrine neoplasia type 2b (men2b), total colectomy

## Abstract

Multiple endocrine neoplasia type 2B (MEN2B) is a rare autosomal dominant disorder caused by RET proto-oncogene mutations, classically associated with medullary thyroid carcinoma (MTC), pheochromocytoma, and gastrointestinal ganglioneuromatosis. Gastrointestinal symptoms, including constipation and megacolon, typically present in infancy or childhood; late-onset presentation is rare. We report a 66-year-old woman with MEN2B, previously treated with total thyroidectomy for MTC and bilateral adrenalectomy for pheochromocytoma, who presented with absolute constipation, abdominal distension, nausea, and vomiting. Imaging demonstrated marked colonic dilatation without obstruction. She underwent emergency total colectomy with end ileostomy, and histopathology confirmed diffuse ganglioneuromatosis. This case represents one of the oldest reported presentations of megacolon in MEN2B and highlights the importance of recognising gastrointestinal features in all age groups to allow timely surgical intervention and optimise patient outcomes.

## Introduction

Multiple endocrine neoplasia type 2 (MEN2) is a rare autosomal dominant disorder caused by germline mutations in the RET proto-oncogene. Clinically, it is classified into two phenotypic variants: MEN2A and MEN2B. Extra-endocrine features, including gastrointestinal (GI) manifestations, are more frequent in MEN2B than in MEN2A [1].

In MEN2B, GI symptoms often appear early in life, with constipation and megacolon during infancy or childhood being common non-specific initial signs [2]. These symptoms arise from diffuse ganglioneuromatosis of the bowel wall, which impairs motility and can lead to progressive colonic dilatation [3]. While medical management may provide temporary relief, definitive treatment is usually surgical resection [4]. Although most cases present in early childhood, chronic megacolon can also present much later in life in patients with MEN2B [3].

## Case presentation

A 66-year-old woman with MEN2B, diagnosed in childhood, presented with absolute constipation for five days, associated with abdominal distension, nausea, and vomiting. She has been on medical treatment for chronic constipation for many years. She had undergone total thyroidectomy for medullary thyroid carcinoma (MTC) and bilateral adrenalectomy for pheochromocytoma in her youth.

On examination, she exhibited marfanoid body habitus, everted eyelids, and prominent lips. The abdomen was grossly distended with audible bowel sounds and diffuse tenderness, but no guarding or rigidity. Laboratory investigations were unremarkable.

**Figure 1:**
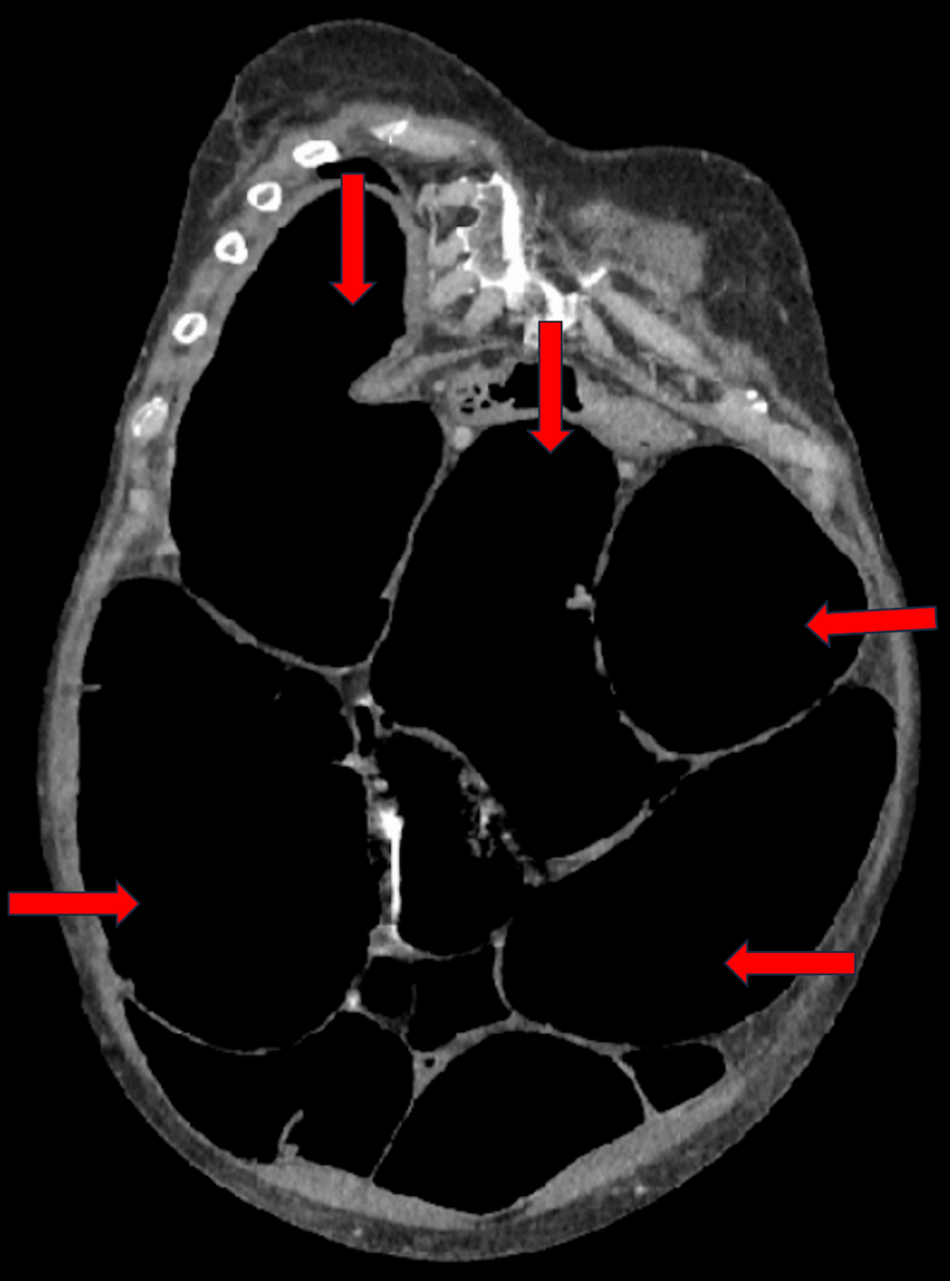
Coronal CT of the abdomen demonstrating multiple loops of large bowel that are significantly dilated (red arrows).

**Figure 2:**
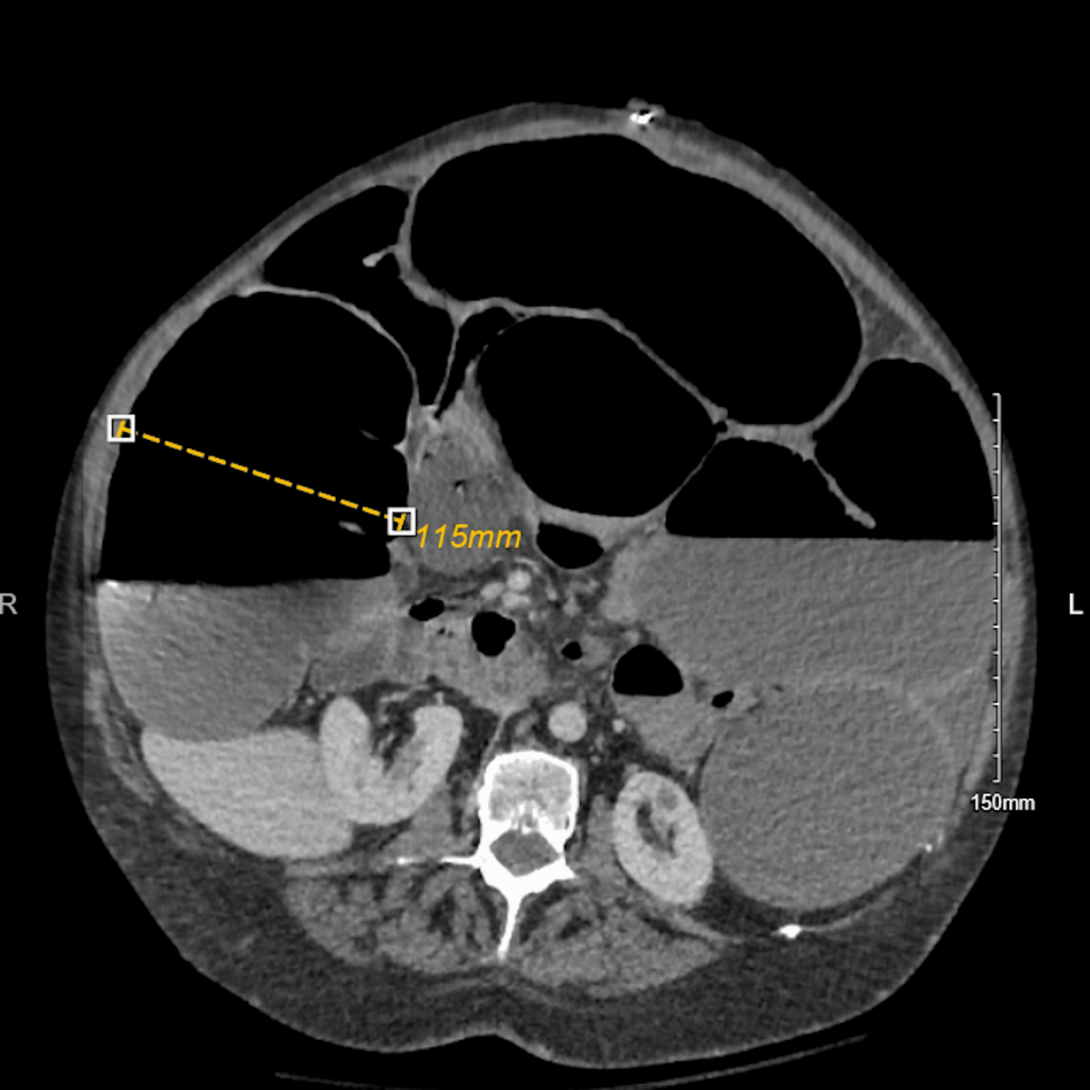
Axial CT of the abdomen demonstrating a markedly distended large bowel loop measuring 11.5 cm, as indicated by the dotted yellow line.

Computed tomography (CT) abdomen and pelvis showed marked colonic dilatation up to 11.5 cm, with loss of bowel tone, and no evidence of perforation or obstructive lesion (Figures 1-2).

She underwent laparotomy, adhesiolysis, and manual bowel decompression. Postoperatively, she developed abdominal distension. Repeat CT abdomen and pelvis showed persistent abdominal distention findings similar to the previous CT scan. Abdominal distention subsequently resulted in midline laparotomy wound dehiscence.

**Figure 3:**
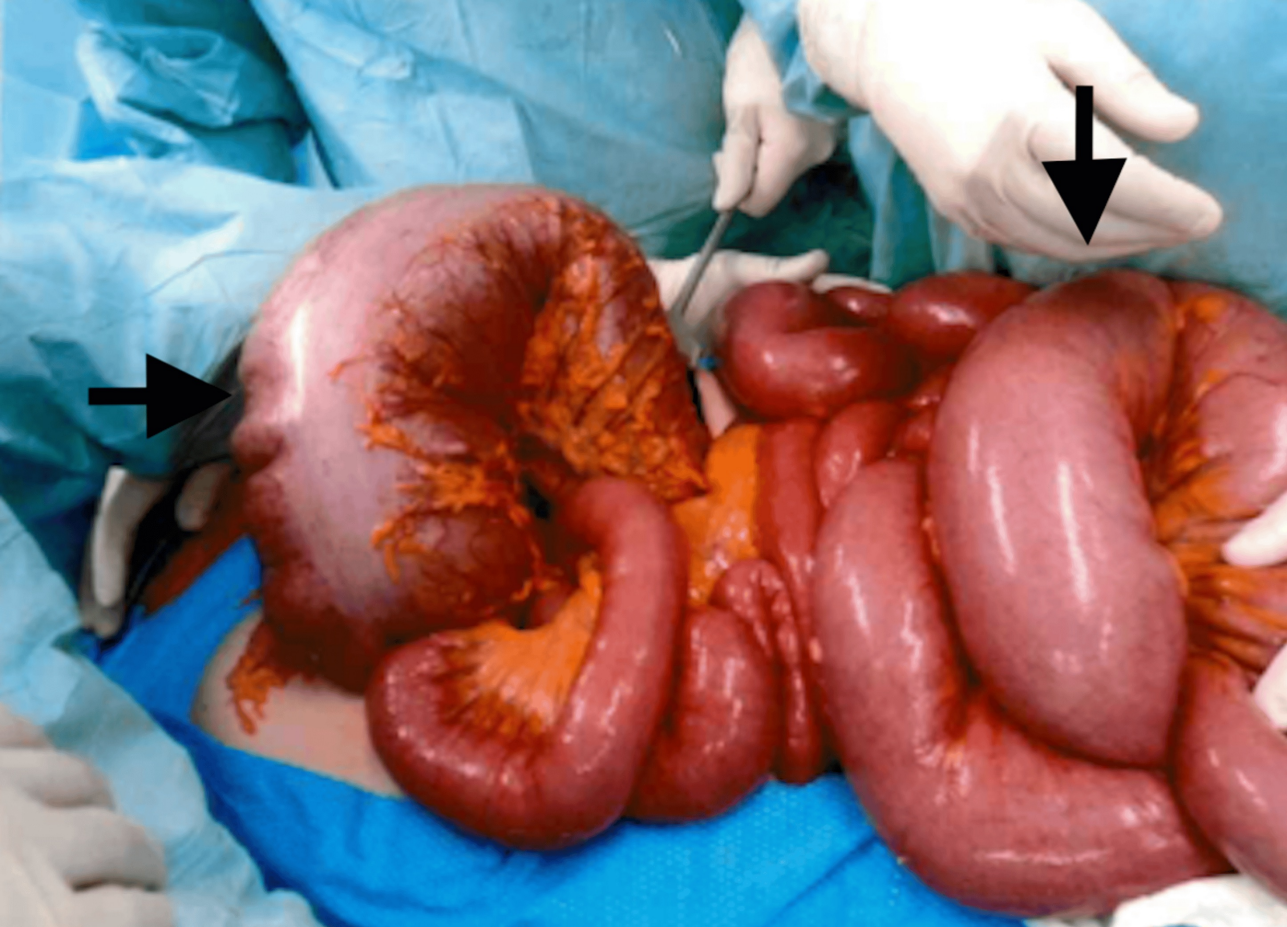
Intraoperative image demonstrating markedly dilated large-bowel loops (black arrows).

**Figure 4:**
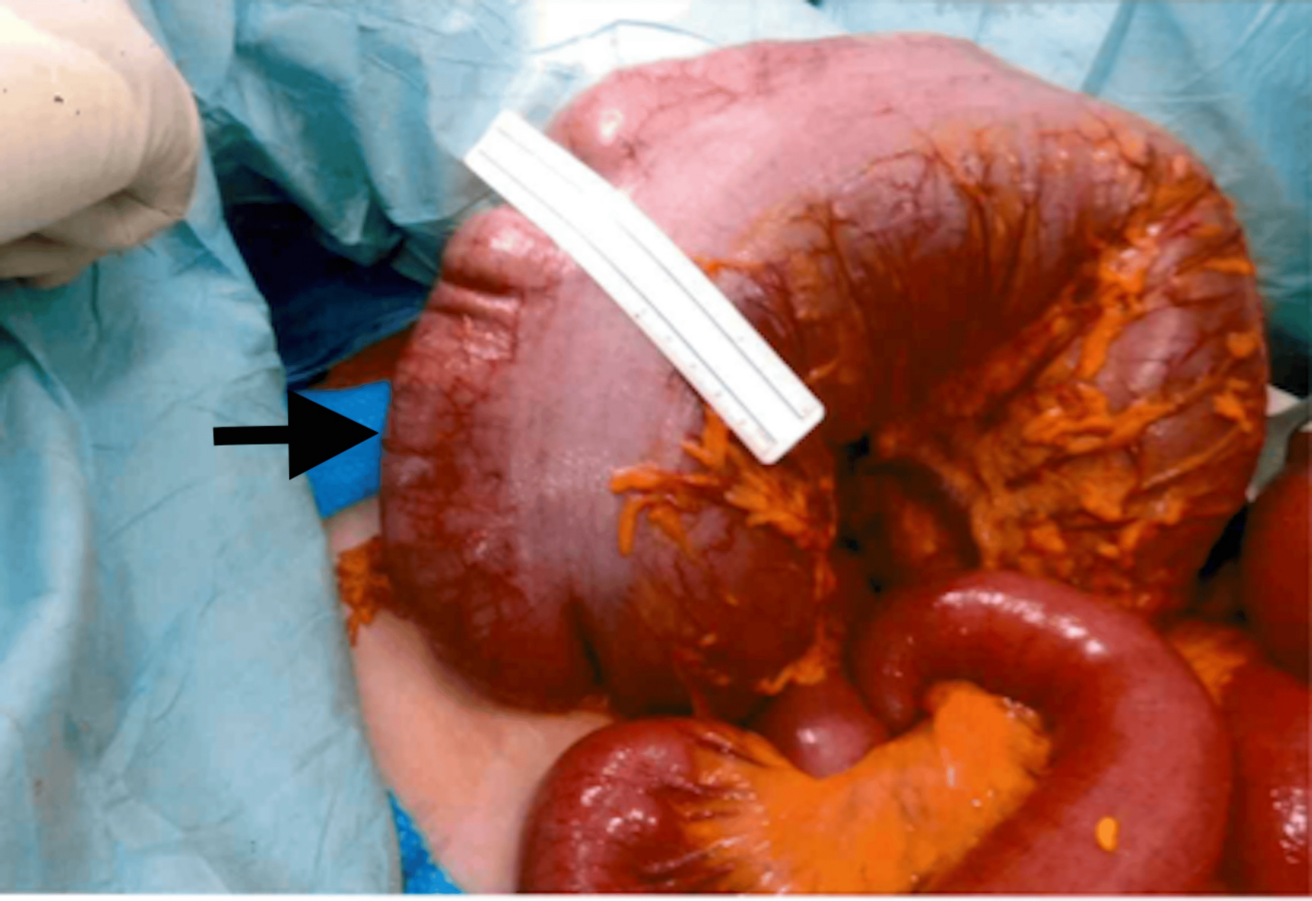
Intraoperative image demonstrating a significantly dilated transverse colon measuring 15 cm (black arrow).

Emergency laparotomy revealed a massively dilated colon without perforation or mechanical obstruction. A total colectomy with end ileostomy was performed (Figures 3-4). Patient’s postoperative recovery was prolonged due to the development of COVID-19 infection, requiring ICU and vasopressor support for the first two days following surgery. She was subsequently weaned off support and transferred out of the ICU once clinically stable. After completing stoma education and rehabilitation, she was discharged on postoperative day 14 to a nursing home facility for further convalescence before eventually returning home.

**Figure 5:**
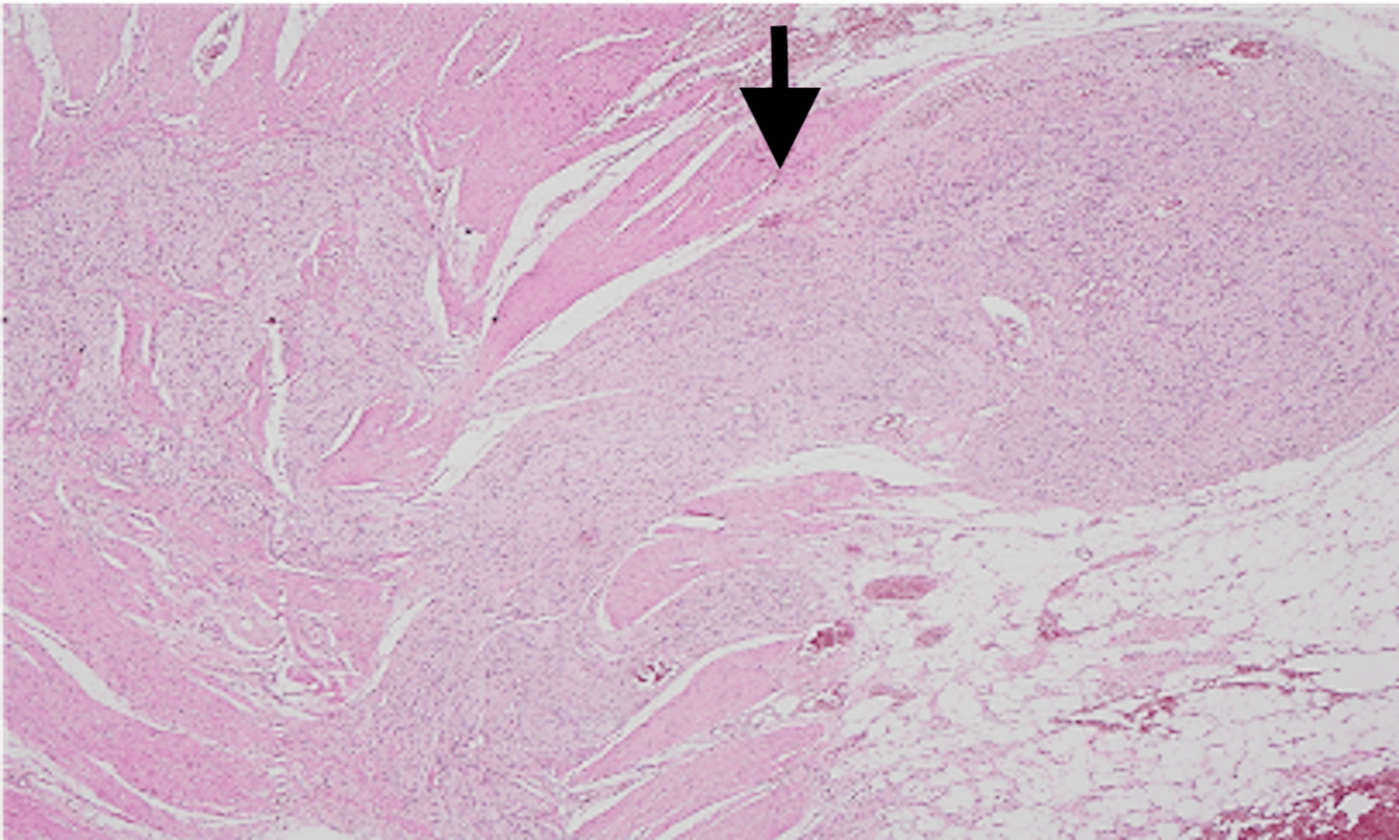
Hematoxylin and eosin (H&E)-stained sample (magnification 2x). The muscularis propria displaying hypertrophied nerve fibers and mature ganglion cells (black arrow).

**Figure 6:**
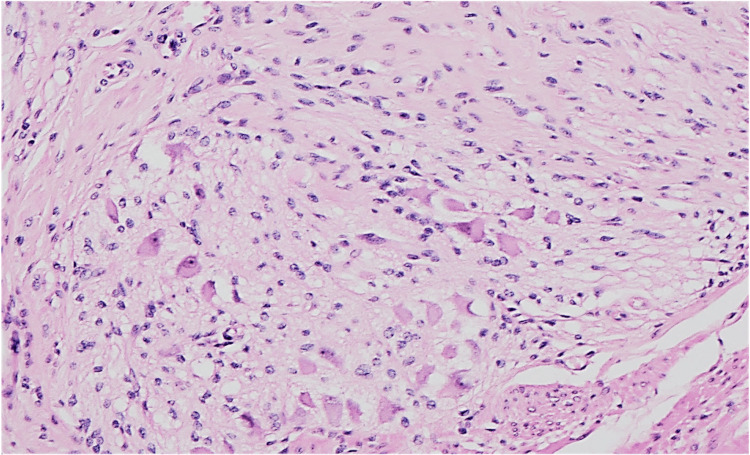
Hematoxylin and eosin (H&E)-stained section at 2× magnification demonstrating a ganglioneuroma composed of hypertrophied nerve fibers and mature ganglion cells.

Gross pathology confirmed marked colonic dilatation without tumour. Histology demonstrated diffuse ganglioneuromatosis with neural hyperplasia and ganglion cell proliferation within the muscularis propria and submucosa, extending to the subserosa (Figures 5-6).

## Discussion

MEN2 is a rare genetic disorder with two main variants: MEN2A and MEN2B. MEN2B is classically associated with MTC, pheochromocytoma, and GI ganglioneuromatosis. The risk of MTC in MEN2B is virtually 100%, and it typically presents in the second decade of life, being the leading cause of mortality in these patients [4]. Pheochromocytoma occurs in approximately 50% of cases, with a mean age of onset around 25 years [4]. Other characteristic features include marfanoid habitus, mucosal neuromas, ophthalmological signs, and skeletal abnormalities, though not all features are present in every patient [5,6].

GI symptoms are common in MEN2B and may present as constipation or megacolon, with constipation often being the earliest manifestation [1,7]. This results from the proliferation of ganglion cells and nerve fibres within the bowel wall, leading to abnormal motility, loss of tone, and progressive dilatation [1]. Diffuse ganglioneuromatosis may mimic Hirschsprung’s disease clinically; however, the key histological distinction lies in the presence of ganglion cells in the former [1]. Radiographically, megacolon is defined as dilatation exceeding 12 cm in the cecum, 8 cm in the ascending colon, and 6.5 cm in the rectosigmoid. In MEN2B, chronic megacolon secondary to diffuse ganglioneuromatosis is typically refractory to medical therapy, with surgical resection being the treatment of choice [3,4].

Given that GI symptoms often precede endocrine manifestations, early recognition of diffuse ganglioneuromatosis in MEN2B provides an important diagnostic window. Prompt diagnosis may enable earlier detection and treatment of MTC, potentially improving survival [3].

While GI involvement usually manifests in infancy or early childhood, late-onset presentation is rare; only a handful of cases have been reported in patients over the age of 50, including one at age 60 [3] and another at age 50 following total thyroidectomy for MTC [3,8]. Our patient’s presentation at age 66 represents one of the oldest known cases described in the literature to date.

This case reinforces the need for clinical vigilance for diffuse ganglioneuromatosis in MEN2B patients of all ages presenting with refractory constipation and colonic dilatation.

## Conclusions

Megacolon in MEN2B is usually an early-life clinical manifestation. Our patient, a known case of MEN2B with a history of MTC and prior thyroidectomy, presented much later in life. Awareness of the GI features of MEN2B and timely intervention are essential, as early recognition and appropriate surgical management remain the mainstay of treatment.
